# GPCRs in Cancer: Protease-Activated Receptors, Endocytic Adaptors and Signaling

**DOI:** 10.3390/ijms19071886

**Published:** 2018-06-27

**Authors:** Aleena K. S. Arakaki, Wen-An Pan, JoAnn Trejo

**Affiliations:** 1 Biomedical Sciences Graduate Program, School of Medicine, University of California, La Jolla, San Diego, CA 92093, USA; aarakaki@ucsd.edu; 2 Department of Pharmacology, School of Medicine, University of California, La Jolla, San Diego, CA 92093, USA; wep007@ucsd.edu

**Keywords:** invasion, metastasis, breast cancer, arrestins, ARRDC3, lysosomes

## Abstract

G protein-coupled receptors (GPCRs) are a large diverse family of cell surface signaling receptors implicated in various types of cancers. Several studies indicate that GPCRs control many aspects of cancer progression including tumor growth, invasion, migration, survival and metastasis. While it is known that GPCR activity can be altered in cancer through aberrant overexpression, gain-of-function activating mutations, and increased production and secretion of agonists, the precise mechanisms of how GPCRs contribute to cancer progression remains elusive. Protease-activated receptors (PARs) are a unique class of GPCRs implicated in cancer. PARs are a subfamily of GPCRs comprised of four members that are irreversibly activated by proteolytic cleavage induced by various proteases generated in the tumor microenvironment. Given the unusual proteolytic irreversible activation of PARs, expression of receptors at the cell surface is a key feature that influences signaling responses and is exquisitely controlled by endocytic adaptor proteins. Here, we discuss new survey data from the Cancer Genome Atlas and the Genotype-Tissue Expression projects analysis of expression of all PAR family member expression in human tumor samples as well as the role and function of the endocytic sorting machinery that controls PAR expression and signaling of PARs in normal cells and in cancer.

## 1. Introduction

G protein-coupled receptors (GPCRs) are a large and diverse family of signaling receptors that function in cancer growth and development by regulating cellular proliferation, invasion, migration, immune cell-mediated functions, angiogenesis and survival at metastatic sites [1,2,3]. In addition, GPCRs are known to function in metastasis [2,3], and there are limited targeted treatment options for patients with metastatic cancer. GPCRs are cell surface receptors with highly druggable sites and the largest class of drug targets, with over 30% of current FDA-approved drugs targeting GPCRs [4,5]. However, despite the success and promise of GPCRs as therapeutic targets, there are currently no drugs in the clinic used for the treatment of cancer that specifically target GPCRs. It is now well recognized that GPCR activity can be altered in cancer through aberrant overexpression, gain-of-function activating mutations, mutations in downstream G protein signaling effectors, and increased production and secretion of GPCR activating ligands by both tumor cells and surrounding stromal cells [6,7,8,9]. Given the broad and diverse functions of GPCRs in cancer, understanding the mechanisms that lead to aberrant GPCR expression and function in tumor progression is important for the development of new effective treatment strategies for metastatic cancer.

Several GPCRs have been implicated in metastatic cancer, including the unique family of protease-activated receptor (PARs). PARs transmit signals to extracellular proteases and respond to coagulant serine proteases such as thrombin. There are four PARs encoded in the mammalian genome. PAR1, the prototype for this family, transmits cellular responses to thrombin, the main effector protease of the coagulation cascade. PAR3 and PAR4 also respond to thrombin, whereas PAR2 is activated by trypsin-like serine proteases but not by thrombin. PARs also respond to proteases released by epithelial cells and various cells in the tumor microenvironment. In fact, PAR1 senses and responds to multiple proteases generated in the tumor microenvironment including thrombin, plasmin and matrix metalloproteinase-1 (MMP-1) [10,11,12]. The zinc-dependent MMP-1 has also been reported to promote tumor growth and invasion through activation of PAR1 [11], providing an important link between tumor-generated metalloproteases and PAR signaling. PARs can promote tumor growth, invasion and metastasis but precisely how PARs contribute to cancer progression has yet to be fully elucidated.

The proteolytic nature of PAR activation, which results in irreversible activation, is distinct from most GPCRs. The prototypical PAR1 is activated by irreversible proteolytic cleavage of the N-terminus, revealing a new N-terminal domain that acts as a tethered ligand that binds intramolecularly to the receptor to elicit transmembrane signaling [13,14]. Once activated, PAR1 signals to distinct heterotrimeric G protein subtypes including G_q_, G_i_ and G_12/13_ and triggers RhoGEF-mediated RhoA signaling, increases in intracellular Ca^2+^, MAP kinase activation and signaling by multiple other effectors [15,16]. In addition, activated PAR1 appears to signal to p38 MAP kinase from endosomes through an atypical pathway mediated by ubiquitin and TGFβ-activated kinase-1 binding protein (TAB) proteins [17]. While activated PAR2 also signals through heterotrimeric G proteins at the plasma membrane, signaling is further propagated at endosomes through PAR2-β-arrestin-dependent activation of MAP kinases [18,19]. Dysregulation of these signaling events may then lead to increased tumorigenesis, invasion and metastasis. However, the defects that engender PAR1 and other GPCRs with the capacity to promote cancer invasion and metastasis are not known. Signaling by PARs is directly linked to expression at the cell surface and is controlled by gene transcription as well as internalization, recycling and lysosomal degradation. Here we discuss a survey of PAR family member expression from the Cancer Genome Atlas (TCGA) and the Genotype-Tissue Expression projects (GTEx) in human tumor samples in various cancer types, using a newly developed online platform, Gene Expression Profiling Interactive Analysis (GEPIA) [20] as well as the different endocytic mechanisms that control PAR expression and signaling.

## 2. PAR Expression in Human Cancer

**Figure 1:**
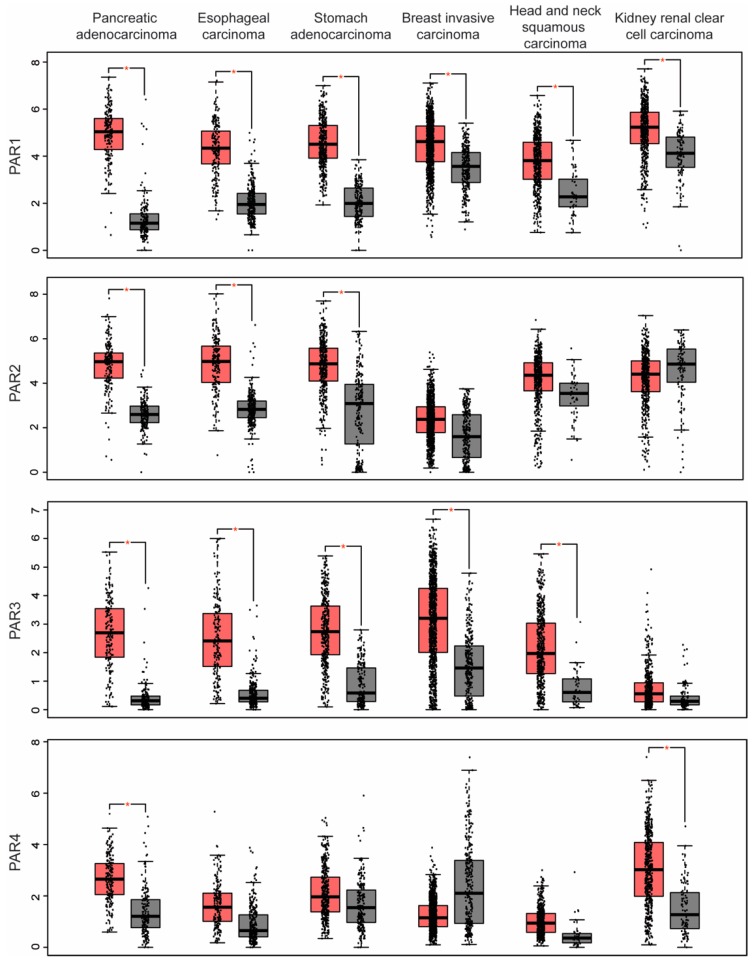
Co-expression of protease-activated receptors in human cancers. GEPIA analysis revealed that multiple PARs are significantly overexpressed in various cancer types. Pancreatic adenocarcinoma (179 tumor, 171 normal) overexpresses all PARs compared to normal tissue; esophageal carcinoma (182 tumor, 286 normal) and stomach adenocarcinoma (408 tumor, 211 normal) overexpress PAR1, PAR2, and PAR3, whereas breast invasive carcinoma (1085 tumor, 291 normal) and head and neck squamous carcinoma (519 tumor, 44 normal) overexpress PAR1 and PAR3 and kidney renal clear cell carcinoma (523 tumor, 100 normal) overexpress PAR1 and PAR4. The RNA-seq data are expressed as relative gene expression using transformed log_2_ (TPM+1) value (*Y*-axis) of tumor (red) and normal (grey) samples from different cancer types and displayed as a whisker plot. The whisker plot solid horizontal black line is the median, the box represents the upper and lower quartiles and the two lines (whiskers) outside the box extend to the highest and lowest observations of the sample population. The difference in PAR expression in tumors compared to normal tissue control is significant based on one-way ANOVA (* *p* < 0.01). TPM, transcript per million.

The TCGA and GTEx projects have yielded RNA-Seq data for tens of thousands of cancer and non-cancer patient samples. These large data sets have provided a unique opportunity to survey PAR upregulation and downregulation of expression as well as co-expression with other PARs in various types of human cancer versus normal patient samples. Using Gene Expression Profiling Interactive Analysis (GEPIA, http://gepia.cancer-pku.cn/), we found that PAR1 and PAR3 are most often upregulated in similar human cancer types including pancreatic adenocarcinoma, esophageal carcinoma, stomach adenocarcinoma, breast invasive carcinoma, head and neck squamous carcinoma and kidney renal clear cell carcinoma (see Figure 1 and Table 1). Interestingly, PAR1 and PAR3 can form heterodimers and PAR3 has been shown to modulate the activity of PAR1 by potentiating its signaling to thrombin [21]. In addition, PAR1–PAR3 heterodimer preferentially interacts with Gα_13_ more than monomeric PAR1 [21], and Gα_13_ signaling is known to be important for progression of certain types of cancers [22].

**Figure 2:**
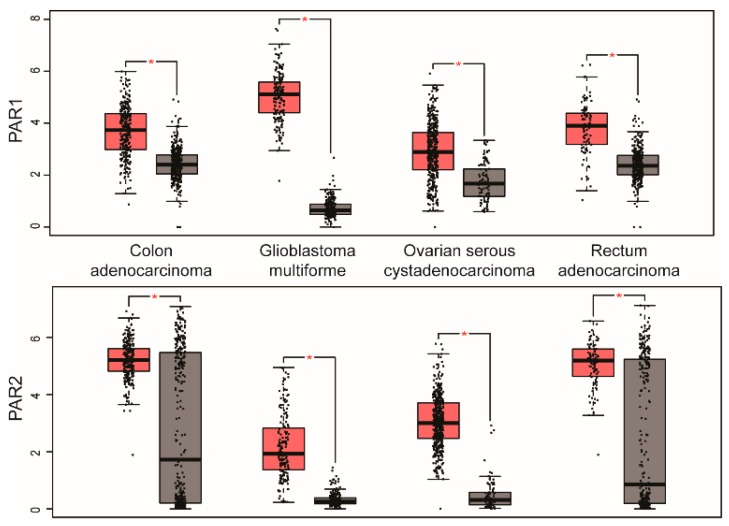
Human cancers with significant PAR1 and PAR2 overexpression. A survey of cancers using GEPIA analysis revealed several cancer types with only PAR1 and PAR2 overexpression compared to normal tissue, including colon adenocarcinoma (275 tumor, 349 normal), glioblastoma multiforme (163 tumor, 207 normal), ovarian serous cystadenocarcinoma (426 tumor, 88 normal), and rectum adenocarcinoma (92 tumor, 318 normal). The RNA-seq data are expressed as the relative gene expression using transformed log_2_ (TPM+1) value (*Y*-axis) of tumor (red) and normal (grey) in different cancer types and displayed as whisker plots as described in Figure 1. The data showed a significant difference in PAR1 and PAR2 expression in tumors compared to normal tissue using one-way ANOVA (* *p* < 0.01).

In contrast, PAR1 is rarely found co-expressed with PAR4 (Figure 1), except in two cancer types pancreatic adenocarcinoma and kidney renal clear cell carcinoma (Table 1). Although both PAR1 and PAR4 have been reported to form heterodimers in studies ectopically expressing the receptors [23], we were unable to demonstrate endogenous PAR1 co-association with endogenous PAR4 in Dami megakaryocytic cells lines compared to robust PAR4 association with the P2Y12 receptor [24]. Interestingly, PAR1 and PAR2 also have a high incidence of co-expression either together with other PARs as observed in pancreatic adenocarcinoma, esophageal carcinoma, stomach adenocarcinoma, and kidney renal clear cell carcinoma (see Figure 1 and Table 1). However, in certain cancer types, such as colon adenocarcinoma, glioblastoma multiforme, ovarian serous cystadenocarcinoma and rectum adenocarcinoma, PAR1 and PAR2 are the only PARs expressed (Figure 2 and Table 1). Unlike other GPCR heterodimer formation, there is substantial evidence that both endogenous and exogenous PAR1 and PAR2 form a functional heterodimer [25]. There is also substantial evidence to suggest that the PAR1 tethered ligand can bind intermolecularly to transactivate PAR2 in COS7 cells and endothelial cells [26]. Thrombin-induced melanoma cell motility and metastasis also appears to require PAR1 transactivation of PAR2 [27], suggesting that PAR1-PAR2 may function together in multiple cancer types to promote tumor progression. 

**Figure 3:**
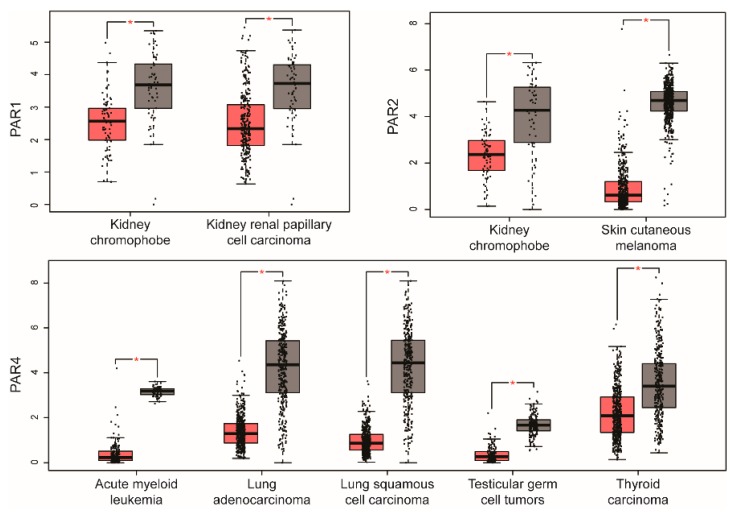
Human cancers with downregulation of PAR1, PAR2 and PAR4 expression. A few cancers displayed significant decreases in expression of PARs based on GEPIA analysis. PAR1 showed significantly downregulation in kidney chromophobe (66 tumor, 53 normal) and kidney renal papillary cell carcinoma (286 tumor, 60 normal). PAR2 was significantly downregulated in kidney chromophobe and skin cutaneous melanoma (461 tumor, 558 normal). PAR4 was significantly downregulated in acute myeloid leukemia (173 tumor, 70 normal), lung adenocarcinoma (483 tumor, 347 normal), lung squamous cell carcinoma (486 tumor, 338 normal), testicular germ cell tumors (137 tumor, 165 normal), and thyroid carcinoma (512 tumor, 337 normal). RNA-seq data are expressed as relative gene expression using transformed log_2_ (TPM+1) value (*Y*-axis) of tumor (red) and normal (grey) samples from different cancer types and displayed as whisker plots as shown in Figure 1. The data showing differences in PAR expression in tumor compared to normal tissue are significant based on one-way ANOVA (* *p* < 0.01).

In addition to PAR upregulation, analysis of PAR expression indicates that certain PARs are downregulated in various cancer types. PAR1 and PAR2 are both significantly downregulated in kidney chromophobe, whereas only PAR1 is downregulated in kidney renal papillary cell carcinoma and PAR2 is only downregulated in skin cutaneous melanoma (see Figure 3 and Table 1). Intriguingly, PAR4 shows greater prevalence of downregulation in five different cancer types, namely acute myeloid leukemia, lung adenocarcinoma, lung squamous cell carcinoma, testicular germ cell tumors and thyroid carcinoma, compared to upregulation detected in only two cancer types (Figure 3 and Table 1). However, the role of downregulation of PAR expression in these distinct cancer types is not known.

In addition to gene transcription, trafficking of PARs is critical for maintaining an appropriate amount of receptor at the cell surface. Once internalized, agonist activated GPCRs are sorted at endosomal membranes by adaptor proteins and are either recycled back to the cell surface or targeted to lysosomes for degradation. Intracellular trafficking of GPCRs has important roles in signal termination, signal propagation from internal compartments and resensitization. Many GPCRs require posttranslational modification with ubiquitin and interaction with ubiquitin-binding domains (UBDs) of the endosomal-sorting complex required for transport (ESCRT) machinery for lysosomal sorting. However, not all GPCRs including PARs require direct ubiquitination or all components of the ESCRT machinery for degradation in the lysosome, suggesting that alternate sorting pathways exist. Below, we discuss the pathways by which PARs are internalized, recycled and/or sorted to lysosomes for degradation and their dysregulation in cancer.

## 3. Internalization and Recycling of PARs and Implications in Cancer

### 3.1. Clathrin-Mediated Endocytosis of PARs

**Figure 4:**
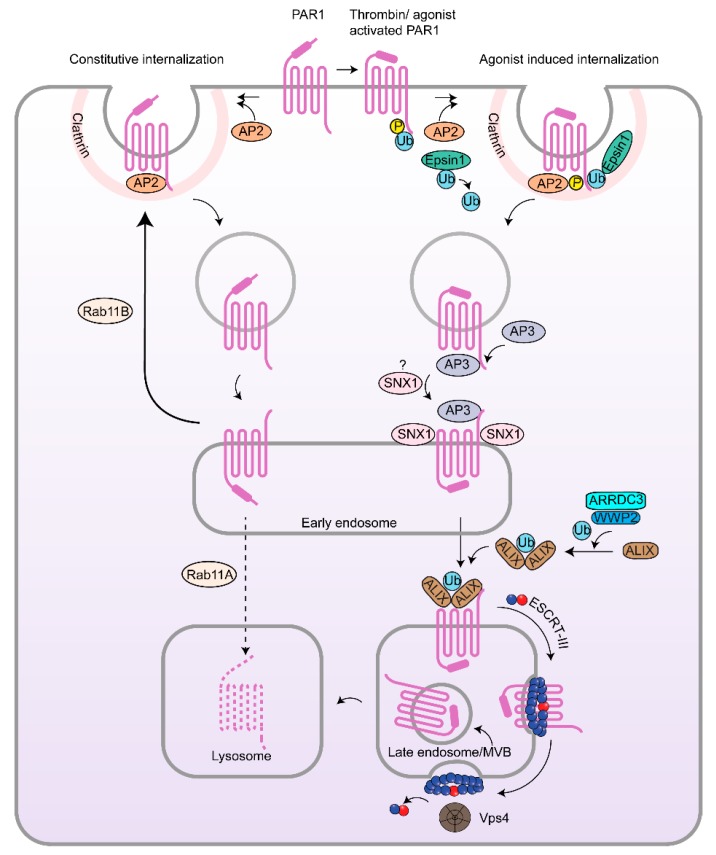
Endocytic trafficking of PAR1. PAR1 undergoes constitutive and agonist-activated internalization induced by thrombin cleavage of the N-terminus or by the peptide agonist SFLLRN. Unactivated PAR1 is constitutively internalized by AP-2 recognition of a distal tyrosine-based motif within the cytoplasmic C-terminus of PAR1. Internalized PAR1 is then sorted to early endosomes and then recycled back to the cell surface via a Rab11B-dependent pathway, whereas a small pool of receptor escapes recycling and is sorted by Rab11A to lysosomes and degraded. In contrast, agonist activation of PAR1 results in rapid phosphorylation and ubiquitination and internalization through a dynamin- and clathrin-dependent pathway mediated by AP-2 and epsin-1. AP-2 binds the phosphorylated distal C-terminus of activated PAR1 rather than the tyrosine-based motif to regulate activated PAR1 internalization. PAR1 activation also promotes epsin-1 deubiquitination, facilitating the ability of epsin-1 to bind activated PAR1 to facilitate internalization. Internalized PAR1 is then sorted sequentially at early endosomes by engaging AP-3 and SNX1 followed by ALIX, which requires ARRDC3 and WWP2-mediated ALIX ubiquitination and dimerization. ALIX, ARRDC3 and WWP2 are essential for targeting PAR1 to intraluminal vesicles of multivesicular bodies (MVBs)/late endosomes via ECSRT-III charged MVB protein 4 (CHMP4) and AAA-ATPase vacuolar protein sorting 4 (Vps4). Degradation of PAR1 in lysosomes is ultimately required for signal termination.

Endocytic trafficking of GPCRs is important for controlling the spatial and temporal dynamics of signaling, and this is particularly relevant for PARs. Of the PAR family members, PAR1, PAR2 and PAR4 are internalized from the plasma membrane through clathrin-mediated endocytosis in most cell types, whereas the process by which PAR3 is removed from the cell surface is not known. Interestingly, PAR1 displays two modes of endocytosis, constitutive and agonist-induced internalization, and both are critical for controlling the fidelity of signaling. Uncleaved, unactivated PAR1 is constitutively internalized from the cell surface to early endosomes and then recycled back to the cell surface (Figure 4) [28]. PAR1 constitutive internalization serves to generate an intracellular pool of uncleaved receptor that can replenish the cell surface with naïve PAR1 to allow for rapid resensitization to thrombin stimulation independent of de novo protein synthesis [29,30,31]. Unlike most classic GPCRs that are dependent on β-arrestins, constitutive internalization of PAR1 is mediated by the clathrin adaptor protein complex-2 (AP-2), a heterotetrameric complex comprised of α, β2, μ2 and σ2 adaptin subunits. The large AP-2 α- and β2-adaptin subunits bind to clathrin, whereas the μ2-adaptin subunit recognizes tyrosine-based sorting motifs and facilitates recruitment of receptor cargo to clathrin-coated pits [32]. We previously showed that the μ2-adaptin subunit directly binds to the PAR1 cytoplasmic tail distal tyrosine-based sorting motif YXXØ, where X is any amino acid proceeding a bulky hydrophobic residue Ø. Both the PAR1 tyrosine-based motif and μ2-adaptin subunit are required for PAR1 constitutive internalization, the generation of an intracellular pool of uncleaved receptor and for facilitating rapid resensitization to thrombin signaling in endothelial cells and HeLa cells [33].

Similar to most GPCRs, agonist-activated PAR1 undergoes rapid phosphorylation and internalization through a dynamin- and clathrin-dependent pathway (Figure 4) [34]. Dynamin is a large GTPase required for scission of clathrin-coated pits from the plasma membrane [35,36]. However, in contrast to most GPCRs, efficient internalization of activated PAR1 requires both AP-2 and the clathrin adaptor protein epsin-1 and neither β-arrestin-1 or β-arrestin-2 isoforms (Figure 4) [34,37,38,39,40]. Although β-arrestins are not essential for PAR1 internalization, activated PAR1 desensitization is severely impaired in β-arrestin-1 knockout mouse embryonic fibroblasts (MEFs) [34], indicating that β-arrestin isoforms serve distinct functions for PAR1. AP-2 and epsin-1 recognize activated PAR1 phosphorylation and ubiquitination, respectively, to promote receptor internalization from the cell surface [37]. Epsin-1 belongs to the epsin protein family that contains ubiquitin-interacting motifs (UIMs) and function in clathrin-dependent endocytosis of ubiquitinated cell surface receptors [41]. Activation of PAR1 induces de-ubiquitination of epsin-1, which may release an intramolecular interaction between the ubiquitin moiety and the UIM of epsin-1, and thereby facilitate the binding of epsin-1 to ubiquitinated PAR1 to facilitate receptor internalization [37]. In addition, activated PAR1 phosphorylation of the distal cytoplasmic C-terminal tail of the receptor is required for AP-2-dependent internalization, rather than the canonical tyrosine-based sorting motif YXXØ [37], which is important for constitutive internalization. These studies indicate that AP-2 utilizes distinct mechanisms to control constitutive versus activated PAR1 internalization.

In addition to phosphorylation and ubiquitination, many GPCRs are also reversibly modified by palmitoylation. GPCR palmitoylation occurs through covalent linkage of palmitic acid, a 16-carbon saturated fatty acid, to juxtamembrane cysteine residues localized within the cytoplasmic tail of GPCRs via a thioester bond [42,43]. Palmitoylation is dynamically regulated by palmitoyl acyltransferases and palmitoyl-protein thioesterases that add and remove the palmitoyl group, respectively [44]. Palmitoylation of GPCRs has been reported to control various functions including endocytic trafficking and signaling [45]. We showed that PAR1 is palmitoylated at highly conserved juxtamembrane cysteine residues within the cytoplasmic tail domain [46]. Interestingly, a palmitoylation deficient PAR1 mutant displays enhanced constitutive internalization and accelerated lysosomal degradation and showed minimal effect on receptor biogenesis, agonist-induced internalization or signaling. Such defective PAR1 palmitoylation attributes result from AP-2 utilization of the proximal tyrosine-based sorting motif YXXØ rather than the distal motif observed under normal conditions [46]. This discrepancy leads to markedly enhanced constitutive internalization of palmitoylation deficient PAR1. Therefore, palmitoylation of PAR1 is necessary for maintaining proper receptor secondary structure and utilization of appropriate tyrosine-based sorting motifs and is important for maintaining appropriate expression of PAR1 at the cell surface.

**Figure 5:**
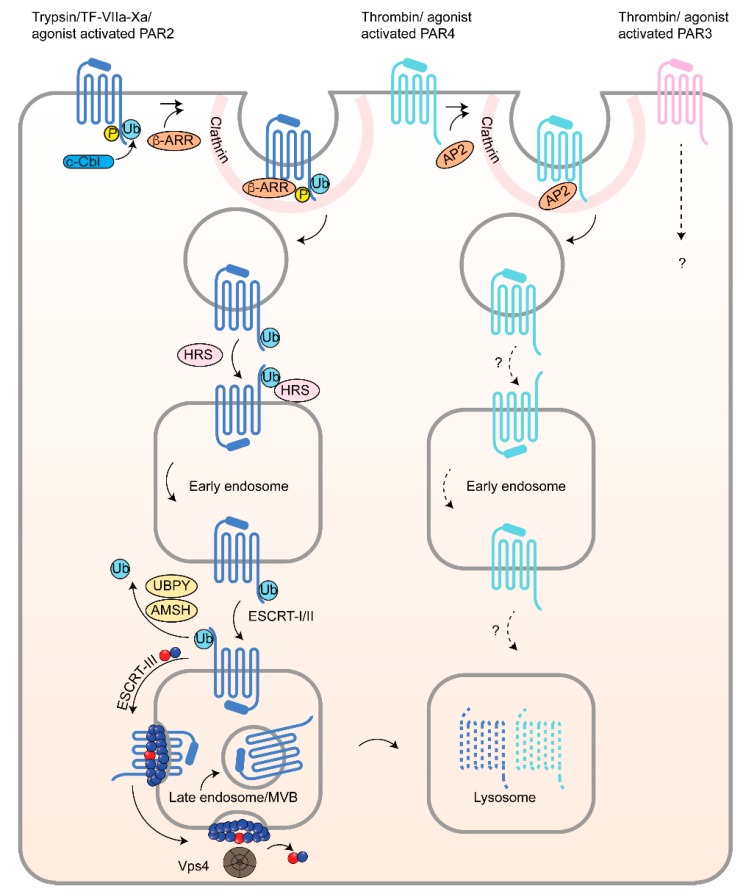
Endocytic trafficking of PAR2, PAR3 and PAR4. Agonist-activation of PAR2 can occur by trypsin, tissue factor (TF)-VIIa-Xa or peptide agonist SLIGKV and induces phosphorylation and ubiquitination followed by receptor internalization, which is mediated by β-arrestins (β-ARR) through a clathrin-dependent pathway. Ubiquitination of activated PAR2 by the E3 ubiquitin ligase c-Cbl, functions primarily in PAR2 endosomal-lysosomal trafficking. Ubiquitinated PAR2 is recognized by HRS at early endosomes and required for lysosomal sorting, after deubiquitination mediated by the AMSH and UBPY deubiquitinating enzymes that enable sorting of PAR2 to MVBs by the ESCRT-III/Vps4 machinery and ultimately degradation. Agonist-activation of PAR4 by thrombin or AYPGKF induces internalization mediated by AP-2 and occurs through a clathrin-dependent pathway similar to PAR1. AP-2 binds activated PAR4 by recognizing a tyrosine-based sorting motif present in the third intracellular loop of the receptor. Internalized PAR4 is then sorted to endosomes and lysosomes for degradation. The endocytic adaptor proteins that facilitate PAR4 endosomal-lysosomal trafficking are unknown. The mechanisms that control thrombin-activated PAR3 internalization and trafficking to lysosomes is not known.

Similar to PAR1, agonist-induced PAR2 and PAR4 internalization occurs through clathrin-mediated endocytosis and is dependent on dynamin (Figure 5) [47,48]. Despite that fact that activated PAR2 is internalized and sorted predominantly to lysosomes like PAR1, PAR2 desensitization and internalization require β-arrestins [18,19,49]. Internalization of activated PAR2 and uncoupling from G protein signaling are both markedly reduced in β-arrestin deficient MEFs [19], like most classic GPCRs. Activated PAR2 is rapidly and robustly phosphorylated on cytoplasmic tail clusters of serine and threonine residues, which is required for β-arrestin recruitment, internalization and desensitization [48]. Although agonist-induced PAR4 is internalized through a dynamin- and clathrin-dependent pathway [47], surprisingly internalization remains intact in β-arrestins knockout MEFs. These findings indicate that β-arrestins are dispensable for activated PAR4 internalization. Rather, agonist-induced internalization of PAR4 requires AP-2 similar to PAR1 (Figure 5). In cells deficient in AP-2 expression, agonist-stimulated PAR4 internalization was virtually abolished in Dami megakaryocytic cells expressing endogenous PAR4 and HeLa cells ectopically expression PAR4 [47]. However, unlike PAR1, the cytoplasmic tail region of PAR4 is dispensable for AP-2-dependent internalization of activated PAR4. Instead, AP-2 utilize a distinct highly conserved YX_3_L tyrosine-based sorting motif present in the third intracellular loop of the receptor [47]. Remarkably, blocking PAR4 internalization by depletion of AP-2 resulted in a significant increase in ERK1/2 signaling but diminished Akt signaling [47]. These studies suggest that endocytic trafficking of PAR4 is tightly linked and essential for controlling proper PAR4 signaling from the cell surface and endosomes. In contrast to other PARs, the mechanisms that regulate PAR3 internalization are not known. Together, these findings provide evidence that not all irreversibly proteolytically activated PARs have evolved to adopt the same sorting machinery and that certain components of the sorting machinery may serve to control signaling functions of the receptors.

### 3.2. β-Arrestins, Epsins and Adaptor Protein Complex-2 (AP-2) and PARs in Cancer

Several clathrin adaptors have been implicated in cancer progression. Here, we discuss the roles of β-arrestins, epsin-1 and AP-2 given their connections to PAR endocytic trafficking. β-arrestin-1 and -2 are multifunctional adaptor molecules that control the magnitude, duration and spatial aspects of GPCR signaling [50]. In cancer, β-arrestins have been reported to mediate tumor growth [51,52], migration [53], invasion and metastasis in multiple cancers including ovarian, colorectal, breast and leukemia [53,54,55,56,57,58,59]. β-arrestins display both increased and decreased expression in multiple human patient tumor types compared to normal control (Table 2). In addition, β-arrestins have been demonstrated to modulate GPCR-driven tumor motility and metastasis. β-arrestin-1 is required for prostaglandin E_2_ (PGE_2_) induced transactivation of epidermal growth factor receptor (EGFR), Akt signaling and colorectal cancer cell motility in vitro and liver metastasis in vivo [54]. In addition, β-arrestin-1 mediates endothelin-A receptor (ET_A_R)-driven ovarian cancer invasion and metastasis by activating β-catenin signaling [56,59]. Interestingly, both β-arrestin-1 and -2 are required for PAR2-stimulated ERK1/2 activation in pseudopodia and induction of invasive breast cancer cell migration via a process that appears to be mediated by constitutive secretion and activation of PAR2 by trypsin-like proteases [53]. In contrast to PAR2, there is no evidence for a role of β-arrestins in PAR1-modulated signaling in tumor progression. Beyond GPCRs, β-arrestins may possess alternative functions in cancer that are mediated by the distinct β-arrestin isoforms. Genetic deletion of β-arrestin-2 but not β-arrestin-1 in mice impaired the progression of chronic myelogenous leukemia (CML) by inhibiting the Wnt/β-catenin pathway [58]. In addition, β-arrestin-1 and -2 have distinct subcellular localizations. β-arrestin-1, but not β-arrestin-2, is detected in both the cytosol and nucleus. The nuclear β-arrestin-1 has been shown to promote tumor progression by regulating gene transcription through interacting with transcription factors including β-catenin [56], HIF1 [57] and E2F1 [60]. It will be important to determine if β-arrestin-1 also translocates to the nucleus in response to PARs stimulation to regulate gene expression for tumor progression.

In addition to β-arrestins, epsins and to a lesser extent AP-2 have been implicated in cancer (Table 2). In prostate cancer, epsins-1 and -2 are up-regulated and depletion or knockdown inhibits tumorigenesis and progression in both human xenograft and spontaneous developing prostate cancer mouse models [61]. In mouse vascular endothelium, depletion of epsin-1 and -2 inhibits tumor growth by impairing the endocytosis and degradation of vascular endothelial growth factor receptor 2 (VEGFR2), leading to excessive VEGF signaling and results in disorganized vasculature and nonproductive tumor angiogenesis [62]. Whether epsins play a role in PARs-mediated tumor angiogenesis and progression remains unclear.

### 3.3. Rab11, PAR Recycling and Implications in Cancer

In general, both constitutive and agonist-stimulated GPCRs are internalized and sorted to endosomes, where GPCRs are dissociated from ligands and dephosphorylated, and then recycled back to the plasma membrane and replenish the cell surface with unactivated receptors important for cellular resensitization. Recycling of internalized receptors back to the plasma membrane occurs through either bulk membrane flow or sequence-dependent recycling via a distinct tubular endosomal microdomain stabilized by the actin machinery [63,64]. Rab11 proteins are a subfamily of small GTPases including Rab11A, Rab11B and Rab11C (also known as Rab25) and together with effector proteins are important for vesicle formation, movement, tethering and fusion and membrane recycling. Rab11 has been implicated in regulating GPCR endocytosis and intracellular trafficking [65,66].

While proteolytically activated PARs are generally precluded from recycling, and rather are directly targeted to lysosomes for degradation for signal termination, we recently discovered that Rab11A and Rab11B play distinct roles in constitutive PAR1 intracellular trafficking (Figure 4) [28]. Although neither Rab11A nor Rab11B is required for PAR1 constitutive internalization, Rab11B siRNA-mediated knockdown decreased PAR1 cell surface expression by blocking PAR1 constitutive recycling to the cell surface, which resulted in enhanced PAR1 lysosomal sorting and degradation in endothelial cells and breast cancer cells [28]. In contrast to Rab11B, Rab11A is not required for PAR1 recycling but rather loss of Rab11A function resulted in an increased accumulation of PAR1 in an intracellular pool. Moreover, Rab11A knockdown restored PAR1 levels in Rab11B depleted cells by impeding lysosomal degradation of PAR1, suggesting distinct functions of Rab11A and Rab11B in constitutive PAR1 lysosomal sorting and recycling. Interestingly, depletion of the autophagy related protein 5 (ATG5) also restored PAR1 normal cell surface levels of expression in Rab11B knockdown cells, suggesting that blocking PAR1 recycling engages the autophagic pathway for PAR1 degradation. The mechanistic details responsible for switching PAR1 degradation via the autophagic pathway are not known.

Unlike PAR1, which exhibits pronounced constitutive internalization to early endosomes and recycling, unactivated PAR2 displays minimal early endocytic accumulation [49,67]. However, this does not preclude the existence of an intracellular pool of unactivated PAR2, since PAR2 has been detected at both the plasma membrane and Golgi apparatus at steady state [49,68]. In this case, agonist-activation of PAR2 initiates recycling of the receptor from the Golgi apparatus to the plasma membrane, a process critical for cellular resensitization. Interestingly, this process is accelerated by overexpression of Rab11A, suggesting that Rab11A has an important role in recycling of PAR2 from the Golgi-enriched receptor pool rather than an early endosomal pool as observed with PAR1 [28,68].

Both Rab11A and Rab11B show significant changes in expression in various human cancer types with a prevalence for Rab11A (Table 2). Other studies further suggest that Rab11A has an oncogenic role in several cancers including colorectal [69], pancreatic [70], breast [71,72], and lung cancers [73]. Rab11A overexpression promotes cellular transformation by regulating E-cadherin turnover in colorectal cancer cells [69]. In pancreatic cancer, Rab11A expression correlates with tumor-node-metastasis stage and promotes cell proliferation, cell cycle progression and invasion through GSK3β/Wnt/β-catenin pathway [70]. In breast cancer cells, downregulation of Rab11A by tumor suppressor miRNA miR-320a and miR-452 inhibits breast cancer growth, motility and invasion [71,72]. In lung cancer, high Rab11A levels correlate with advanced stage and poor survival [73]. Rab11A promotes non-small cell lung cancer (NSCLC) cell aggressiveness in vitro and tumor growth in vivo through upregulation of YAP [73]. These results suggest that Rab11A may regulate tumor progression through vesicle trafficking-dependent and -independent mechanisms. While there is minimal knowledge about the role of Rab11A in GPCR-driven cancer progression, Rab11A has been shown to recruit Gβγ to endosomes and sustain lysophosphatidic acid (LPA) activated Akt signaling, which is associated with LPA-induced cell survival [74]. Whether Rab11A plays a key role in modulating the compartmentalized signaling of GPCRs and/or PARs to regulate tumor progression remains to be investigated.

## 4. Lysosomal Sorting of PARs and Dysregulation in Cancer

Due to the irreversible proteolytic cleavage of PARs, which results in the generation of a tethered ligand that cannot diffuse way (Figure 4), signaling by protease-activated receptors is tightly regulated. Once activated, PARs are internalized from the plasma membrane and sorted to lysosomes for degradation, a process critical for ultimately terminating signaling [75,76]. However, studies indicate that perturbation of the endocytic trafficking machinery in cancer results in slowed PAR degradation and/or recycling of activated receptors back to the cell surface that signal persistently [77,78,79]. Here, we discuss the endocytic adaptors and mechanisms responsible for lysosomal sorting of PARs and the implications in tumor progression.

### 4.1. Endosomal Sorting of PAR1 by Sorting Nexin 1 (SNX1) and Adaptor Protein-3 (AP-3) and Role in Cancer

Early studies of GPCR lysosomal sorting revealed a classic role for ubiquitination and canonical endosomal sorting complexes required for transport (ESCRTs) [80]. However, not all GPCRs require direct ubiquitination and canonical ESCRTs for lysosomal sorting [81]. We showed that activation of a ubiquitin-deficient PAR1 mutant sorted directly to intraluminal vesicles (ILVs) of multivesicular bodies (MVBs)/lysosomes and degraded similar to wildtype receptor (Figure 4) [38,82], and raised the question of how a GPCR can be targeted to lysosomes for degradation independent of ubiquitination.

SNX1 belongs to a group of diverse trafficking proteins that contain a PX domain (phospholipid-binding motif) and a carboxyl-terminal coiled-coil domain [83,84]. Given that the yeast functional homolog of SNX1 mediates vacuolar sorting [85], a role for SNX1 in regulation of lysosomal sorting of PAR1 in mammalian cells was examined. We found that activated PAR1 co-associated and co-localized with endogenous SNX1 on endosomes [86]. Disruption of SNX1 function with a deletion mutant or siRNA-mediated depletion inhibited agonist-induced PAR1 lysosomal degradation, whereas internalization remained intact, indicating a critical role for SNX1 in sorting of PAR1 from early endosomes to lysosomes (Figure 4) [86,87]. SNX1 is known to interact with components of the mammalian retromer complex and hepatocyte growth factor-regulated tyrosine kinase substrate (Hrs), a component of ESCRT-I. However, activated PAR1 degradation was not affected by depletion of retromer Vps26/Vps35 subunits or the ubiquitin-binding ESCRT components Hrs or Tsg101, an Hrs-interacting protein, indicating that agonist-induced PAR1 lysosomal degradation occurs independent of retromer activity and the canonical ESCRT machinery (Figure 4) [87]. In addition to PAR1, SNX1 was found to interact with at least ten distinct GPCRs in vitro, suggesting a broad role for SNX1 in regulating trafficking of other GPCRs [88].

Several studies suggest that SNX1 may function as a tumor suppressor in colorectal and non-small cell lung cancer (NSCLC). In a study of 20 colon cancer patients, 75% had significantly less SNX1 protein expression compared to matched adjacent normal controls while a separate cohort of patients showed decreased SNX1 mRNA in 18 of 19 tumor samples [89]. Depletion of SNX1 expression by shRNA in colorectal cell lines resulted in increased cell proliferation, decreased apoptosis and susceptibility to anoikis [89]. A more recent study showed that SNX1 protein and mRNA transcripts are markedly decreased in colorectal cancer tissues from 237 patients compared to paired non-cancerous tissues and the down-regulation of SNX1 protein was strongly associated with poor overall survival rate of colorectal cancer patients [90]. Ectopic SNX1 expression also prevented cell growth and increased drug sensitivity of tumor cells [90]. MicroRNA miR-95 has also been shown to directly downregulate SNX1 in colorectal and NSCLC [91,92,93]. MiR-95 is upregulated in colorectal and NSCLC patient tumor samples as well as in corresponding cell lines and shown to promote cell growth and tumorigenicity [91,92]. These studies indicate that loss of SNX1 expression in colorectal and NSCLC, promotes tumor progression. However, it is also possible that SNX1 overexpression perturbs endocytic sorting contributes to cancer progression (Table 2). In both cases, disruption of SNX1 expression is likely to perturb SNX1-driven tubule subdomain stabilization important for receptor endocytic sorting [63].

The adaptor protein complex-3 (AP-3) is known to localize to budding sites of tubule-sorting endosomes containing lysosomal-associated membrane proteins [94] and is also important for PAR1 lysosomal sorting. The μ3-adaptin subunit of the tetraheteromeric AP-3 complex recognizes tyrosine-based motifs to facilitate cargo sorting [95]. We found that sorting of PAR1 from early endosomes to lysosomes is initiated by AP-3 binding to a tyrosine-based YXXL motif localized within the C-tail of PAR1 (Figure 4) [96]. In addition, a PAR1 tyrosine mutant which cannot bind to AP-3 fails to sort to lysosomes and depletion of AP-3 blocks both wildtype and ubiquitin-deficient PAR1 lysosomal degradation [96]. These findings support the idea that PAR1 sorts to lysosomes via a SNX1 and AP-3-dependent pathway independent of ubiquitination. AP-3 has also been shown to regulate the trafficking of several other mammalian GPCRs. In mouse neuroblastoma cells, depletion of AP-3 results in increased cell surface expression of the cannabinoid receptor-1 (CB1R), suggesting a role for AP-3 in endosome to lysosome sorting or retention in endosomes [97]. The neuronal specific AP-3 isoform also directly interacts with the M5 muscarinic acetylcholine receptor and functions in regulating M5 recycling in neurons [98]. While AP-3 expression is increased in various types of cancer (Table 2), its functional role in cancer progression has yet to be determined.

### 4.2. Atypical Ubiquitin-Independent Lysosomal Sorting of PAR1 Mediated by ALIX and ARRDC3

Similar to cargo lysosomal sorting through the canonical ESCRT pathway, PAR1 is sorted to lysosomes through an atypical pathway that requires sequential interactions with distinct endocytic adaptor proteins. After sorting by AP-3 and SNX1, activated PAR1 directly interacts with the adaptor protein ALG-2-interacting protein X (ALIX) [82,96]. In fact, AP-3 is required for facilitating PAR1 interaction with ALIX, as knockdown of AP-3 and a PAR1 tyrosine-based motif mutant with impaired AP-3 binding fails to bind to ALIX following agonist stimulation [96]. These findings indicate that PAR1 is targeted to a distinct lysosomal pathway mediated by AP-3 (Figure 4). ALIX expression is also essential for agonist-induced PAR1 lysosomal degradation [82]. PAR1 contains a highly conserved YPX_3_L motif localized within intracellular loop 2 that directly interacts with the central V domain of ALIX (Figure 4) [82]. ALIX has also been shown to bind to the cytoplasmic tail domains of other GPCRs, including the vasopressin V2R and D1-like and D3 dopamine receptors and regulates receptor trafficking [99,100]. In these examples, however, the receptors lack classic ALIX binding YPX_n_L motifs and thus ALIX may function in an ancillary role to facilitate receptor trafficking. Besides PAR1, seven other mammalian GPCRs were found to contain conserved YPX_n_L motifs within their second intracellular loop, including the adrenoreceptor α1_B_, angiotensin receptor AT2, galanin receptor GAL_2_, histamine receptor H_2_, neuropeptide FF receptor NPFF2, neuropeptide S receptor NPS, and purinergic receptor P2Y_1_ [82]. Of this subset of GPCRs, only the P2Y_1_ receptor has been studied and shown to use a ubiquitin-independent and ALIX-dependent lysosomal sorting pathway like PAR1 [101], suggesting that this pathway is broadly applicable to multiple GPCRs.

While it is known that intracellular trafficking of cell surface receptors is important for regulating the magnitude, duration and spatial aspects of cell signaling, emerging studies also suggest that signaling by the receptors themselves function in a reciprocal manner to modulate the endocytic machinery [102]. Consistent with this idea, we found that ALIX activity is regulated through agonist-activated PAR1 stimulated signaling that leads to WWP2-mediated ubiquitination of ALIX, dimerization and enhanced activity at sorting PAR1 to MVBs/lysosomes (Figure 4) [103]. Importantly, the α-arrestin domain-containing protein-3 (ARRDC3) is responsible for recruitment of the WWP2 HECT-domain containing E3 ubiquitin ligase to ALIX and subsequent ubiquitination [103]. ARRDC3 is a member of the mammalian α-arrestin family that shares similar domain homology with mammalian β-arrestins, which have important and diverse roles in GPCR trafficking [104]. However, unlike β-arrestins, ARRDC3 lacks a polar core, essential for β-arrestin binding to activated and phosphorylated GPCRs and additionally contains a C-terminal PPxY motif that binds to WW domains of HECT-domain containing E3 ubiquitin ligases [105,106,107]. These findings suggest that β-arrestins and α-arrestins likely serve distinct functions. We showed that ARRDC3 co-associates and colocalizes with activated PAR1 [103]. In addition, ARRDC3 expression is required for agonist-induced PAR1 interaction with ALIX and lysosomal degradation [103]. Together, these studies provide substantial evidence for the existence of an atypical ALIX and ARRDC3-dependent lysosomal sorting pathway for a subset of mammalian GPCRs.

ARRDC3 has been identified as a tumor suppressor in breast and prostate cancer [108,109,110,111] (Table 2). ARRDC3 expression is low or absent in the highly aggressive basal-like breast cancer [108], and associated with tumor grade, metastasis and recurrence. Moreover, ARRDC3 localizes to a gene cluster on chromosome 5 deleted in 17% of basal-like breast cancers compared to 0% deletion in luminal breast cancers [108,109]. ARRDC3 expression has also been shown to be suppressed through epigenetic silencing and small non-coding micro-RNAs [110,112,113]. Unlike other endocytic adaptor molecules, the mechanism by which ARRDC3 functions as a tumor suppressor has been investigated as described below.

In invasive breast cancer, a target of ARRDC3 is the integral membrane protein integrin β4 [109], which is enriched in triple-negative breast cancer and a marker of poor prognosis [109]. In more recent work, we found that loss of ARRDC3 expression is also responsible for defective PAR1 trafficking in invasive breast cancer [79], suggesting that ARRDC3 tumor suppressor function is linked to both integrin β4 and GPCR trafficking. We previously showed that invasive breast carcinoma cells exhibit dysregulated PAR1 lysosomal degradation and persistent signaling, which promotes cellular invasion and tumor growth [77,78]. Since ARRDC3 expression is either lost or suppressed in invasive breast cancer, we employed a lentiviral induction system to restore ARRDC3 expression in MDA-MB-231 cells. Strikingly, we found that re-expression of ARRDC3 is sufficient to restore normal activated PAR1 lysosomal sorting [79]. In contrast to ARRDC3, ALIX expression in invasive versus non-invasive breast cancer is variable and is consistent with human cancers that exhibit both upregulated and downregulated ALIX expression (Table 2). In human cancers, ARRDC3 expression is also suppressed in breast, kidney, ovarian and pheochromocytoma, while other cancers clearly show increased expression. The effect of increased ARRDC3 expression in these specific cancer types is not known. Similar to studies in HeLa and endothelial cells, ALIX was shown to be required for ARRDC3-mediated degradation of activated PAR1 in invasive breast carcinoma (Figure 4) [79]. We showed previously that defective PAR1 lysosomal trafficking results in recycling of “activated” receptor back to the cell surface and persistent signaling and increases cellular invasion and tumor growth [77,78]. As expected, ARRDC3 re-expression attenuated persistent signaling by activated PAR1 as well as PAR1-mediated cellular invasion [79]. This study is the first to identify an important role of the ARRDC3 endocytic adaptor protein that functions as a tumor suppressor by regulating GPCR trafficking and signaling in invasive breast cancer.

Modulating the ubiquitination status of proteins can clearly regulate the function of oncoproteins by causing gain-of-function or of tumor suppressor proteins that result in loss-of-function. RING and HECT-domain E3 ubiquitin ligases mediate the covalent attachment of ubiquitin to substrate proteins, whereas deubiquitinating enzymes are responsible for removing ubiquitin moieties from proteins. The HECT-domain containing WWP2 E3 ligase ubiquitinates many proteins and has been implicated in regulating the activity of tumor promoters and suppressors in different cancer types. WWP2 appears to regulate the expression of the well characterized tumor suppressor phosphatase and tensin homolog (PTEN) [114,115] in endometrial cancer [116] and squamous cell carcinoma [117]. In squamous cell carcinoma, WWP2-mediated loss of PTEN accelerated tumor cell growth and activation of the PI3K/AKT signaling pathway. However, depletion of WWP2 decreased cell growth, blocked PI3K/AKT signaling, increased PTEN expression, promoted cell cycle arrest, and inhibited tumor growth in vivo [117]. A study in thyroid cancer cells showed that depletion of hypoxia-inducible factor-1α (HIF-1α) resulted in a decrease in WWP2 expression, suggesting a possible mechanism by which hypoxic conditions in the tumor may result in increased WWP2 expression [118]. WWP2 also regulates Notch3 ubiquitination and attenuates signaling resulting in cell cycle arrest in ovarian cancer [119]. In invasive breast carcinoma, it is not known if WWP2 expression is altered and thereby contributes to the dysregulation of PAR1 trafficking.

### 4.3. ESCRT-III/Charged MVB Protein 4 (CHMP4) and Vps4-Dependent Sorting of PAR1

Although the ESCRT-0/I machinery Hrs and Tsg101, and receptor ubiquitination, are not necessary for agonist-induced PAR1 lysosomal degradation [38,87], the ultimate uptake of PAR1 into ILVs of MVBs/lysosomes requires ESCRT-III and Vps4, an AAA-ATPase for receptor degradation (Figure 4) [82]. CHMP4 is a subunit of ESCRT-III, which lacks ubiquitin-binding domains and functions as a mediator of intraluminal vesicle scission [120]. Depletion of the CHMP4B and CHMP4C isoforms by siRNA in HeLa cells prevented agonist-induced degradation of PAR1 [82]. We also showed that CHMP4 interacts with activated PAR1 and ALIX, via its Bro domain, and facilitates CHMP4B interaction with activated PAR1 [82]. Additionally, ARRDC3 is critical for ALIX interaction with CHMP4B through its recruitment of WWP2 and subsequent ubiquitination of ALIX by WWP2 [103]. WWP2 siRNA depletion also inhibited agonist-induced ALIX and CHMP4B interaction, which is necessary for PAR1 lysosomal degradation [103]. Vps4 catalyzes ESCRT-III disassembly and recycling [121]. We used a catalytically inactive variant of Vps4 and observed a blockade of agonist-induced PAR1 degradation [82], indicating that CHMP4 and Vps4 function are necessary for the final steps of PAR1 incorporation into MVBs/lysosomes.

In addition to human tumor samples that show largely upregulation of CHMP4B expression in various cancer types (Table 2), CHMP4B expression was also found to be high in human patient hepatocellular carcinoma tissue compared with adjacent normal tissue and high CHMP4B expression correlated with poor survival [122]. In addition, CHMP4B siRNA depletion in hepatocellular carcinoma cells reduced cell proliferation and sensitized cells to doxorubicin [122]. In breast cancer, the Vps4A homolog Vps4B transcript mRNA levels were down-regulated in higher grade or recurrent tumors compared with lower grade tumors [123].

### 4.4. PAR2 Lysosomal Sorting Requires Ubiquitination and Canonical ESCRT

In contrast to PAR1, lysosomal sorting of activated PAR2 requires posttranslational modification with ubiquitin and the canonical ubiquitin-binding ESCRT machinery (Figure 5) [124], similar to other classic GPCRs. A ubiquitin-deficient PAR2 mutant signaled and internalized from the cell surface after agonist stimulation; however, mutant PAR2 was retained in early endosomes, failed to transit to lysosomes and was not degraded [38,124]. Unlike most GPCRs, ubiquitination of PAR2 is mediated by the RING-finger E3 ubiquitin ligase c-Cbl that has a predominant role in ubiquitination of receptor tyrosine kinases [125,126,127,128]. A mutant c-Cbl lacking the RING-finger domain disrupted agonist-induced PAR2 ubiquitination, lysosomal degradation and enhanced signaling [124].

Although controversial, the literature does indicate that c-Cbl most likely acts as a tumor suppressor. The loss of c-Cbl expression has been documented in many different types of cancer, whereas only a few cancers show c-Cbl overexpression [129]. In addition, many downstream targets of c-Cbl including EGFR, c-MET, JAK2 exhibit increased expression that correlate with tumorigenic phenotypes [129].

As noted above, PAR2 expression is upregulated in multiple types of cancer (Figure 1 and Figure 2 and Table 1) and thus c-Cbl may function directly or indirectly to control aspects of PAR2 expression in addition to regulating other tumor drivers. Once ubiquitinated by c-Cbl, PAR2 engages Hrs, a ubiquitin binding component of the canonical ESCRT machinery [130,131]. Disruption of Hrs function resulted in loss of agonist-induced PAR2 lysosomal trafficking and degradation, however activated PAR2 internalization remained intact [132]. In addition, overexpression of Hrs also resulted in dysregulated agonist-induced PAR2 trafficking, suggesting any changes in expression level of Hrs may lead to dysregulation in its function. Unlike other PARs, two deubiquitinating enzymes, UBPY and AMSH, have been shown to be important for removal of ubiquitin from PAR2 prior to uptake by MVBs/lysosomes [133]. Interestingly, immunohistochemistry of tumor biopsies from stomach, colon, liver, cervix and melanoma patients showed increased expression of Hrs protein [134]. This study also showed that inhibition of Hrs resulted in decreased cell colony formation and inhibited tumorigenesis and metastasis, due to increased E-cadherin expression and decreased β-catenin signaling [134], suggesting that Hrs may promote tumor progression through multiple mechanism including modulating GPCR function.

In contrast to PAR1 and PAR2, the mechanisms that regulate PAR3 and PAR4 trafficking are understudied and poorly characterized. We recently showed that, upon agonist stimulation, PAR4 is internalized through clathrin-coated pits and then sorted to early endosomes and then lysosomes [47]. However, the mechanism that mediate PAR4 and PAR3 lysosomal sorting have yet to be elucidated.

## 5. Conclusions

Even though GPCRs are superior drug targets, and there is urgent need for newer, better effective therapies for treatment of metastatic cancer and other aspects of cancer progression, it is remarkable that certain GPCRs have not advanced as potential therapeutic targets. Here, we focus this review on the expression and function of PARs in various types of cancer. Given the unique mechanism of proteolytic activation, the precise regulation of PAR surface expression is critical for controlling the fidelity of signaling. We generated a new survey of data from the Cancer Genome Atlas and the Genotype-Tissue Expression projects and unveiled interesting PAR expression and co-expression patterns that correlate with potential function. In addition, endocytic mechanisms are important for governing the magnitude, duration and spatial aspects of signaling by PARs. New studies reveal that particular endocytic adaptor proteins that control PAR trafficking and signaling have also been implicated in various types of cancer. While our knowledge of the mechanism that control PAR1 and PAR2 and to a lesser extent PAR4 trafficking is substantial, virtually nothing is known about how PAR3 trafficking is controlled. In addition, the new findings of expression of PARs across different cancer types derived from RNA seq data is provocative, yet the mechanisms that lead to aberrant overexpression of PAR mRNA transcripts and the potential implications of PAR co-expression with other PARs in cancer are not known and important to determine in future studies.

## Figures and Tables

**Table 1:** Protease-activated receptor (PAR) expression in human cancers. Gene Expression Profiling Interactive Analysis (GEPIA, http://gepia.cancer-pku.cn/), a newly developed online platform using RNA sequencing expression data from the Cancer Genome Atlas (TCGA) and the Genotype-Tissue Expression projects (GTEx) was used to compare tumor versus normal samples in numerous cancer types. The table summarizes the types of cancers that exhibit either upregulation or downregulation of PARs, compared to normal tissue. ND (not detected).

PARs	Cancers with Upregulated PARs	Cancers with Downregulated PARs
PAR1	Breast invasive carcinoma Colon adenocarcinoma Lymphoid neoplasm diffuse large B-cell lymphoma Esophageal carcinoma Glioblastoma multiforme Head and neck squamous cell carcinoma Kidney renal clear cell carcinoma Brain lower grade glioma Ovarian serous cystadenocarcinoma Pancreatic adenocarcinoma Rectum adenocarcinoma Stomach adenocarcinoma Thymoma	Kidney chromophobe Kidney renal papillary cell carcinoma β
PAR2	Cervical squamous cell carcinoma and endocervical adenocarcinomaColon adenocarcinoma Esophageal carcinoma Glioblastoma multiforme Acute myeloid leukemia Lung adenocarcinoma Lung squamous cell carcinoma Ovarian serous cystadenocarcinoma Pancreatic adenocarcinoma Prostate adenocarcinoma Rectum adenocarcinoma Stomach adenocarcinoma Testicular germ cell tumors Uterine corpus endometrial carcinoma Uterine carcinosarcoma	Kidney chromophobe Skin cutaneous melanoma
PAR3	Breast invasive carcinoma Esophageal carcinoma Head and neck squamous cell carcinoma Pancreatic adenocarcinoma Stomach adenocarcinoma	ND
PAR4	Kidney renal clear cell carcinoma Pancreatic adenocarcinoma	Acute myeloid leukemia Lung adenocarcinoma Lung squamous cell carcinoma Testicular germ cell tumors Thyroid carcinoma

**Table 2:** Expression of endocytic adaptors associated with PAR trafficking in human cancers. The expression of the key endocytic adaptors implicated in PAR trafficking was analyzed using the GEPIA database tool. The table is a summary of the different types of cancers that display an upregulation or downregulation of various endocytic adaptors compared to normal tissue. ND, not detected.

Adaptors	Cancers with Upregulated Adaptors	Cancers with Downregulated Adaptors
PDCD6IP (ALIX)	Glioblastoma multiforme Brain lower grade glioma Pancreatic adenocarcinoma	Adrenocortical carcinoma Uterine corpus endometrial carcinoma Uterine carcinosarcoma
STAMBP (AMSH)	Cholangio carcinoma Lymphoid neoplasm diffuse large B-cell lymphoma Glioblastoma multiforme Brain lower grade glioma Lung squamous carcinoma Pancreatic adenocarcinoma Stomach adenocarcinoma Thymoma	ND
AP2A1 (AP-2)	Pancreatic adenocarcinoma	Testicular germ cell tumor Uterine corpus endometrial carcinoma
AP3B1 (AP-3)	Cholangio carcinoma Lymphoid neoplasm diffuse large B-cell lymphoma Glioblastoma multiforme Brain lower grade glioma Liver hepatocellular carcinoma Pancreatic adenocarcinoma Thymoma	ND
ARRDC3	Glioblastoma multiforme Brain lower grade glioma Pancreatic adenocarcinoma	Breast invasive carcinoma Kidney chromophobe Ovarian serous cystadenocarcinoma Pheochromocytoma and paraganglioma
ARRB1 (β-arrestin-1)	Acute myeloid leukemia	Adrenocortical carcinoma Bladder urothelial carcinoma Cervical squamous cell carcinoma Glioblastoma multiforme Kidney chromophobe Lung squamous carcinoma Uterine corpus endometrial carcinoma
ARRB2 (β-arrestin-2)	Kidney chromophobe Kidney renal clear cell carcinoma Acute myeloid leukemia Pancreatic adenocarcinoma	Adrenocortical carcinoma Bladder urothelial carcinoma Lung adenocarcinoma Lung squamous cell carcinoma Thymoma
CBL	Cholangio carcinoma Brain lower grade glioma Pancreatic adenocarcinoma	Testicular germ cell tumors Uterine corpus endometrial carcinoma
CHMP4B	Cholangio carcinoma Lymphoid neoplasm diffuse large B-cell lymphoma Liver hepatocellular carcinoma Pancreatic adenocarcinoma Thymoma	ND
EPN1 (Epsin-1)	Lymphoid neoplasm diffuse large B-cell lymphoma Thymoma	Testicular germ cell tumor
HGS (Hrs)	Cholangio carcinoma Pancreatic adenocarcinoma Thymoma	Testicular germ cell tumors
RAB11A	Cholangio carcinoma Colon adenocarcinoma Lymphoid neoplasm diffuse large B-cell lymphoma Glioblastoma multiforme Ovarian serous cystadenocarcinoma Pancreatic adenocarcinoma Prostate adenocarcinoma Rectal adenocarcinoma Stomach adenocarcinoma Thymoma	Acute myeloid leukemia
RAB11B	Cholangio carcinoma Thymoma	ND
SNX1	Glioblastoma multiforme Brain lower grade glioma Pancreatic adenocarcinoma Thymoma	ND
TSG101	Cholangio carcinoma Lymphoid neoplasm diffuse large B-cell lymphoma Glioblastoma multiforme Brain lower grade glioma Pancreatic adenocarcinoma Thymoma	ND
USP8	Brain lower grade glioma Pancreatic adenocarcinoma Thymoma	ND
WWP2	Cholangio carcinoma Acute myeloid leukemia	Uterine corpus endometrial carcinoma Uterine carcinosarcoma
